# Harms and benefits of cervical cancer screening among non-attenders in Switzerland: The transition towards HPV-based screening

**DOI:** 10.1016/j.pmedr.2022.101929

**Published:** 2022-07-30

**Authors:** Rosa Catarino, Pierre Vassilakos, Patrick Petignat, Christophe Combescure

**Affiliations:** a Department of Paediatrics, Gynaecology and Obstetrics, Geneva University Hospitals, Boulevard de la Cluse 30, 1205 Geneva, Switzerland; b Geneva Foundation for Medical Education and Research, Route de Ferney 150, 1211 Geneva, Switzerland; c Division of Clinical Epidemiology, Geneva University Hospitals, Rue Gabrielle-Perret-Gentil 4, 1205 Geneva, Switzerland

**Keywords:** Cervical cancer screening, Developed countries, Harms, HPV testing, Screening frequency, Self-HPV

## Abstract

Human papillomavirus (HPV) testing is replacing cytological screening for cervical cancer. Our aim was to assess the expected benefits and harms of different cervical screening strategies. This study is sub-analysis of a previous cost-effectiveness study with a target population of unscreened women without cervical cancer aged ≥ 25 years. A recursive decision-tree with one-year cycles was used to model the life-long natural HPV history. Markov cohort simulations were used to assess the expected outcomes from the model. The outcomes of three strategies were compared with the absence of screening: HPV-testing on self-collected vaginal samples (Self-HPV) followed by colposcopy (Self-HPV/colpo), Self-HPV and triage with cytology (Self-HPV/PAP), cytology and triage with HPV (PAP/HPV). All screening strategies resulted in reductions in cancer cases and deaths. Self-HPV strategies were associated with a lower cancer incidence and mortality life-long, not only when performed every 3 years but also when Self-HPV was performed every 5 years vs cytology every 3 years. The gain in life expectancy obtained was 82 days with Self-HPV/colpo, 81 days with Self-HPV/PAP and 75 days with PAP/HPV compared to no screening. The number of lifetime total visits was greater with PAP/HPV compared with the Self-HPV strategies (13.13 vs < 3). The number of conizations remained relatively stable with the change of screening frequency and strategy. Self-HPV may represent a reasonable balance of harms and benefits when performed every 5 years compared to cytology every 3 years. Self-HPV/PAP yielded the most efficient harm to benefit ratio when using colposcopy as a proxy for harms.

## Highlights

• We evaluate the expected benefits and harms of cervical cancer screening strategies.
• Primary self-sampling with HPV was associated with a lower cancer incidence and mortality compared to cytology.
• Primary self-sampling with HPV resulted in a reduction of screening and follow-up visits.
• Primary self-sampling with HPV has a reasonable balance of harms and benefits when performed every 5 years.

## 1 Introduction

In addition to the cost-effectiveness perspective, every screening programme requires the balancing of benefits and harms. Benefits of cervical cancer screening include the early detection and treatment of cervical pre-cancer, resulting in a reduction in cervical cancer incidence and mortality. Since the early 2010s, primary HPV testing has emerged (Arbyn et al., 2012, Murphy et al., 2012, Ronco et al., 2014) as a more sensitive test to detect precancerous lesions, leading countries to change from cytology-based screening programmes to an HPV-based strategy. Other advantages of HPV primary screening include the possibility to self-collect vaginal samples (Self-HPV) at home with a test performance to detect pre- and cancerous lesions similar to physician-collected samples (Petignat et al., 2007). By avoiding a gynaecological consultation, a previous study has shown that the self-collection strategy may reach the unscreened population in Switzerland, which is still evaluated at about 15 % (Burton-Jeangros et al., 2017, Viviano et al., 2017). Moreover, the predictive value of a negative HPV test at 5 years is greater than the predictive value of a normal Pap test result 3 years after testing (Ronco et al., 2014).

The current Swiss guidelines recommend cytological screening every 3 years for women aged 21–29 years and cytological or HPV screening every 3 years for women aged 30–70 years, as part of a non organized screening system depending on women’s and physician’s initiative (Tirri et al., 2018).

One of the downsides of primary HPV testing is the need for a triage strategy, owing to limited specificity, that can comprise cytology and genotyping, followed by colposcopy (Cuschieri et al., 2018). In consequence, an increased number of referrals for colposcopy has been demonstrated in some prospective and retrospective studies (Farnsworth et al., 2020, Loopik et al., 2021, Ronco et al., 2006, Thomsen et al., 2021, Zorzi et al., 2013). The higher rate of colposcopy examinations is just one potential harm that patients might be exposed to during cervical screening. Many studies have shown that screening-related diagnostic and therapeutic events can result in physical and psychological repercussions for patients (Drolet et al., 2012, Habbema et al., 2017, Korfage et al., 2012, Sharp et al., 2009). Psychological consequences include adverse effects of labelling or early diagnosis and anxiety associated with a false positive test (Maissi et al., 2004, Marteau et al., 1990, McCaffery et al., 2010). Concerning physical symptoms, different events have been described after a Pap test, punch biopsy, or a conisation, including lower abdominal pain, urinary discomfort, feeling sick or dizzy, painful sexual activity, and bleeding or vaginal discharge (Habbema et al., 2017). In addition, there is evidence suggesting that the treatment of pre-cancerous lesions may adversely affect obstetric outcomes such as preterm birth, perinatal mortality, and low birth weight (Arbyn et al., 2008, Kyrgiou et al., 2006). A greater number of colposcopies might also lead to more conisations; additionally, “too much medicine”, comprising overdiagnosis and overtreatment, may result in some other unknown harm for patients.

Even though the benefits of cervical screening outweigh the harm at a population level, this may not be true for each individual woman. Potential harm and benefit might be perceived differently by different groups of women. Younger women might be more concerned about obstetric issues than older women and the latter might be more resistant to extended frequency of screening, which might reflect long-held beliefs about the importance of annual Pap testing together with limited knowledge of the potential harm of over-screening (Ogden et al., 2020, Van der Meij et al., 2019). Women from different economic and educational backgrounds also have different views on cervical screening, with women of lower educational level presenting more concern about the costs of the screening programme itself than other potential harms (van der Meij et al., 2019). Therefore, taking into account individual patient preferences, needs, and values, while respecting changing national guidelines is important. In order to do this, physicians should better inform patients regarding the benefit but also the potential harm of cervical screening, while selecting information that might actually be of greatest importance from the perspective of the women themselves.

We have previously published a cost-effectiveness analysis, which showed that Self-HPV was more cost-effective than cytology-based screening when offered to non-attenders for cervical cancer screening in Switzerland (Vassilakos et al., 2019). This analysis constituted the data set for further analyses in the latter study. Though Self-HPV strategies seem to be associated with higher rate of colposcopy examinations in the literature, our aim was to expose different harms or benefits of different screening strategies, including Self-HPV, among unscreened women through the use of mathematical simulation models. These results could be of assistance to physicians in order to better counsel their patients.

## 2 Methods

This study is a sub-analysis of a previous cost-effectiveness study with a target population of unscreened women, aged ≥ 25 years, living in Switzerland, and without cervical cancer (Vassilakos et al., 2019).

### 2.1 Strategies modelled:

The following four strategies were compared using a life-long decision-based analytical model:

*I) No screening.*

*II) Screening strategy 1: Self-HPV and triage with Pap cytology* (Self-HPV/PAP). Self-HPV testing was performed every 3 years starting at age 25 years. Physician-performed liquid-based cytology (Pap test) to triage HPV-positive women, followed by colposcopy for women with atypical squamous cells of undetermined significance or worse cytology (ASC-US + ); conization if a cervical intraepithelial neoplasia grade 2 or worse (CIN2 + ) was identified through biopsy; Self-HPV was performed after 12 months in cases of cervical intraepithelial neoplasia grade 1 (CIN 1), negative colposcopy, or negative biopsy. HPV and Pap tests from the same sample (liquid medium) were used as tests of cure at 12 months after treatment. If the HPV and Pap tests were negative, screening was performed every 3 years thereafter. In contrast, if the HPV and/or Pap test were positive, a new colposcopy was scheduled. An alternative simulation was performed by increasing the frequency of screening to every 5 years.

*III) Screening strategy 2: Self-HPV followed by colposcopy* (Self-HPV/colpo). This strategy skipped the step of triage with a Pap test, with a view of minimizing the number of clinical visits and to meet the needs of women who risked the most to be lost to follow-up. Self-HPV testing was scheduled every 3 years starting at age 25 years. Colposcopy was performed on HPV-positive women; conization was performed if CIN2+ was identified on biopsy samples; Self-HPV was performed after 12 months in cases of CIN 1, a negative colposcopy, or a negative biopsy. HPV and Pap tests from the same liquid sample were used as tests of cure after 12 months. If the HPV and Pap tests were negative, screening was resumed after every 3 years. In contrast, if the HPV and/or Pap test were positive, a new colposcopy was scheduled. An alternative simulation was performed by increasing the frequency of screening to every 5 years.

*IV) Screening strategy 3 (the currently used strategy): Pap cytology and triage with HPV* (PAP/HPV). A Pap test was scheduled every 3 years starting at age 25 years. Women with ASC-US underwent HPV testing for triage, which was performed on the same liquid medium-based sample. HPV positive women were referred for colposcopy while HPV negative women returned for a routine test after 3 years. Women with a high-grade squamous intraepithelial lesion (HSIL), ASC-H (atypical squamous cells-cannot exclude HSIL), or atypical glandular cells (AGC), were referred for colposcopy. A conization was proposed if CIN2+ was identified at biopsy. In cases of CIN 1, negative colposcopy, or negative biopsy, a Pap test was performed after 12 months. HPV and Pap tests from the same liquid samples were used as tests of cure at 12 months after treatment. If the HPV and Pap tests were negative, women resumed routine screening every 3 years. In contrast, if the HPV and/or Pap test were positive, a new colposcopy was scheduled.

The compliance with each screening procedure was assumed to be 70 %. All screening tests were proposed until the age of 70 years. If FIGO I to IV cervical cancer was detected, appropriate treatment was planned.

### 2.2 Screening Outcomes:

The model was used to generate a number of outcomes for each of the four screening strategies, reflecting both health benefits and harms over the lifetime of screening starting at age 25 years. Harms included total number of Pap and HPV tests (including screening, and surveillance), colposcopies, conizations, and total number of visits. Benefits included detected cancers through screening, cervical cancer cases and deaths prevented, and life-years gained.

### 2.3 Model:

The model used for this analysis has been described in our previous publication (Vassilakos et al., 2019). We constructed a type-specific HPV Markov decision model using estimates of the natural history of HPV, cervical pre-cancer, and cervical cancer. The health states captured were: well, HPV infected, CIN1, CIN2, CIN3, FIGO I to IV, death. A cervical cancer (FIGO stages I to IV) could be detected from symptoms and the patient was then treated. Women staying 5 years with an undetected cervical cancer were considered cured and were no more at risk of cervical cancer. We obtained the annual mortality from another cause than cervical cancer, according to age, from the Swiss Federal Statistical Office.

We calibrated the model assuming that 90 % of women will be HPV infected in their lifetime. This model was used for the strategy with no screening. For the other strategies, screening procedures were introduced in this model.

### 2.4 Analytical methods:

The transition probabilities used in the model were derived from Canfell et al. (Canfell et al., 2004) and Myers et al. (Myers et al., 2000), or were adjusted to a Swiss context. A recursive decision-tree with one-year cycles was used. At each cycle, the cohort was distributed in the health states for the next cycles according to the health of the current cycle and proportionally to the transition probabilities of the current cycle. A probabilistic analysis was conducted to account for the uncertainty on the estimated positivity rates of Self-HPV and PAP tests. Five thousand sets of positivity rates were randomly generated. These distributions by health state were derived from estimated positivity rates reported in Zhao et al (Zhao et al., 2012) and Bigras et al (Bigras and De Marval, 2005): the logit of positivity rates were assumed normally distributed. The standard deviations of these gaussian distributions were the standard error of the estimated logit of the positivity rates. For each of the five thousands sets of positivity rates, the model was assessed using Markov cohort simulations and the expected outcomes were assessed. The 0.025 and 0.975 quantiles were assessed to provide a 95 % credible interval around the expected outcomes. In addition, a one-way sensitive analysis was conducted to check the robustness of the findings in regards of the assumptions on the values of the key parameters.

This modelling study did not require ethical approval since it comprises information freely available in the public domain (Canfell et al., 2004, Myers et al., 2000, Vassilakos et al., 2019) and no patient-related individual data were used.

## 3 Results

### 3.1 Incidence of cervical cancer and mortality (Table 1)

In the absence of screening, 20.4 per 1000 women had a cervical cancer in their lifetime. Most cervical cancer occurred after age 50 years. Almost half of women with a cervical cancer died because of the cancer. The cancer-related mortality was lower in women developing a cervical cancer after 55 years because of deaths related to other causes. The life expectancy for a 25-years old woman was 58.7 years.

Compared with the absence of screening, any of the 3-years frequency screening strategies were associated with a lower incidence of cancer at any age (Table 1). The lifetime incidence of cancer was the lowest with the Self-HPV/colpo strategy: it decreased by 0.21 cervical cancers per 1000 women (95 % CI 0.08 to 0.52) compared with the Self-HPV/PAP strategy and by 1.63 cervical cancers per 1000 women (95 % CI 0.47 to 4.06) compared with the PAP/HPV strategy. In women with a cervical cancer, the cancer-related mortality was decreased. This decrease was observed at any age. The gain in life expectancy compared with the absence of screening obtained was 82 days with Self-HPV/colpo, 81 days with Self-HPV/PAP and 75 days with PAP/HPV.

### 3.2 Cervical cancer detection

In the absence of screening, 72 % of cervical cancers were detected, all through the presence of symptoms, and only 28 % of detected cervical cancers were at FIGO I stage (Table 2). On average, a cervical cancer was detected 3.5 years after its development. With 3-years frequency screening strategies, cervical cancers were detected more frequently, especially in women < 55 years, and at an earlier FIGO stage. The delay between the occurrence and the detection of a cervical cancer was 2.3 years with Self-HPV/colpo, 2.4 years with Self-HPV/PAP, and 2.6 years with PAP/HPV. Around 40 % of cervical cancers in women aged 25–55 years were detected by symptoms using the PAP/HPV strategy and around 30 % with the other strategies.

**Table 1:** Incidence of cervical cancer (per 1000 women) and cancer-related mortality (% of women with a cervical cancer).

	Screening strategies			
	No screening	Self-HPV/colpo	Self-HPV/PAP	PAP/HPV
Incidence of cancer per 1000 women				
Lifetime	20.39	1.85 (1.52 to 2.60)	2.06 (1.66 to 3.06)	3.48 (2.51 to 5.93)
By time of cancer occurrence				
25 to 34 years	1.57	0.48 (0.42 to 0.62)	0.54 (0.46 to 0.70)	0.75 (0.60 to 1.02)
35 to 44 years	3.03	0.29 (0.21 to 0.47)	0.34 (0.24 to 0.59)	0.68 (0.44 to 1.22)
45 to 54 years	3.76	0.20 (0.14 to 0.33)	0.23 (0.16 to 0.42)	0.50 (0.31 to 1.03)
≥55 years	12.03	0.89 (0.75 to 1.18)	0.95 (0.79 to 1.35)	1.54 (1.16 to 2.66)
Cancer-related mortality per 1000 women				
Lifetime	9.73	0.55 (0.44 to 0.84)	0.63 (0.49 to 1.04)	1.19 (0.77 to 2.35)
Cancer-related mortality in % of women with a cancer				
Lifetime	47.7	29.8 (28.7 to 32.5)	30.4 (29.0 to 34.1)	34.3 (30.8 to 39.7)
By time of cancer occurrence				
25 to 34 years	52.8	31.0 (29.5 to 34.3)	32.1 (30.2 to 36.4)	36.2 (31.9 to 42.4)
35 to 44 years	52.4	30.3 (28.8 to 33.9)	30.9 (28.7 to 35.4)	36.0 (31.5 to 42.1)
45 to 54 years	52.0	29.9 (28.4 to 33.5)	30.7 (28.6 to 35.2)	35.6 (31.1 to 41.7)
≥55 years	44.6	28.9 (28.2 to 30.8)	29.2 (28.3 to 31.8)	32.3 (29.8 to 36.8)

95% Credible Intervals are reported in brackets.

**Table 2:** Detection of cervical cancer (percentage of cervical cancer detected, FIGO stage at the time of detection and mode of detection) and cancer-related mortality in women with a detected or an undetected cervical cancer.

	3-years frequency screening strategies			
	No screening	Self-HPV/colpo	Self-HPV/PAP	PAP/HPV
Cancer detection (% of women with a cervical cancer)*				
Lifetime	71.7	75.4 (74.2 to 76.7)	76.3 (75.2 to 77.3)	77.3 (76.4 to 77.4)
By time of cancer occurrence				
25 to 34 years	77.1	89.6 (87.7 to 90.4)	88.9 (86.6 to 90.0)	86.6 (83.1 to 89.0)
35 to 44 years	77.0	89.3 (87.5 to 90.1)	89.0 (86.6 to 90.1)	86.3 (82.9 to 88.7)
45 to 54 years	76.5	89.2 (87.3 to 90.0)	88.8 (86.3 to 89.9)	86.1 (82.6 to 88.5)
≥55 years	68.1	60.3 (58.2 to 63.6)	61.6 (59.3 to 65.2)	66.0 (63.3 to 68.5)
FIGO stage at the time of cancer detection in % of women with a detected cancer				
FIGO 1	28.4	55.3 (51.9 to 56.8)	54.8 (50.0 to 56.9)	49.8 (42.0 to 54.9)
FIGO 2	30.6	26.7 (26.1 to 27.9)	27.0 (26.2 to 28.4)	28.3 (26.9 to 29.7)
FIGO 3	33.0	15.3 (14.5 to 17.1)	15.5 (14.4 to 18.2)	18.4 (15.5 to 23.3)
FIGO 4	7.9	2.7 (2.5 to 3.1)	2.7 (2.5 to 3.4)	3.5 (2.7 to 4.9)
Delay between cancer occurrence and cancer detection, years (mean)	3.51	2.33 (2.26 to 2.49)	2.36 (2.27 to 2.58)	2.59 (2.37 to 2.93)
Mode of detection in % of women with a detected cancer				
Symptoms				
Lifetime	100	44.8 (43.1 to 49.6)	45.1 (42.3 to 52.5)	53.1 (45.2 to 67.9)
By time of cancer occurrence				
25 to 34 years	100	28.4 (24.8 to 37.3)	31.3 (26.3 to 42.7)	43.0 (31.9 to 62.8)
35 to 44 years	100	30.6 (27.1 to 39.1)	31.9 (27.1 to 42.9)	44.7 (33.7 to 64.0)
45 to 54 years	100	30.2 (26.8 to 38.7)	31.9 (27.1 to 42.9)	44.3 (33.4 to 63.6)
≥55 years	100	69.6 (67.9 to 72.0)	67.7 (65.8 to 70.1)	68.1 (66.2 to 76.3)
Cancer-related mortality per 1000 women				
Death with an undetected cancer	5.77	0.45 (0.39 to 0.61)	0.49 (0.41 to 0.70)	0.79 (0.58 to 1.40)
Death with a detected cancer	14.61	1.39 (1.13 to 1.99)	1.57 (1.25 to 2.36)	2.69 (1.93 to 4.53)
Cancer-related mortality in % of women with a cancer				
Undetected cancers	75.8	49.9 (46.6 to 57.2)	52.5 (48.3 to 61.1)	62.2 (54.5 to 71.1)
Detected cancers	36.6	23.2 (22.5 to 25.0)	23.6 (22.6 to 26.0)	26.2 (23.7 to 30.0)

95% Credible Intervals are reported in brackets.

* Percentage of women with a detected cervical cancer, with the number of cancers detected in a lifetime as the numerator and the number of cancers in a women’s lifetime as the denominator.

In women with a non-detected cervical cancer (by symptoms or screening strategy): 76 % died because of the cancer in the absence of a screening strategy; 50 % with Self-HPV/colpo; 53 % with Self-HPV/PAP; and 62 % with PAP/HPV. In women with a detected cervical cancer, the cancer-related mortality was 37 % in the absence of a screening strategy, 23 % with Self-HPV/colpo, 24 % with Self-HPV/PAP, and 26 % with PAP/HPV.

### 3.3 Screening strategy harms and benefits according to screening frequency

Table 3 represents the lifetime benefits and number of tests and treatments for each screening strategy according to a screening frequency from 3 years (base case scenario) to 5 years.

**Table 3:** Screening strategy lifetime harms and benefits for a screening frequency varying from 3 years (base case scenario) to 5 years.

Screening strategy	Screening test and treatment	Frequency of screening tests	
		3 years	5 years
**Self-HPV/colpo**	**Benefits**		
	% of detected cancers (lifetime)	75.4 (74.2 to 76.7)	78.1 (77.7 to 78.5)
	% of detected cancers before 55 years	89.4 (87.5 to 90.2)	86.2 (84.4 to 86.9)
	Mortality	0.55 (0.44 to 0.84)	1.03 (0.83 to 1.52)
	**Harms (Nb of tests and treatment)**		
	Screening test (Self-HPV)	11.03 (10.82 to 11.16)	7.12 (7.01 to 7.19)
	Surveillance (Self-HPV)	1.80 (1.23 to 2.66)	1.20 (0.83 to 1.77)
	PAP + HPV after conization	0.16 (0.14 to 0.17)	0.14 (0.12 to 0.15)
	*Colposcopy*	1.96 (1.39 to 2.83)	1.35 (0.97 to 1.92)
	*Conization*	0.16 (0.14 to 0.17)	0.14 (0.12 to 0.15)
	*Total Visits*	2.29 (1.70 to 3.15)	1.63 (1.24 to 1.63)
**Self-HPV/PAP**	**Benefits**		
	% of detected cancers	76.3 (75.2 to 77.3)	78.3 (77.5 to 78.8)
	% of detected cancers before 55 years	88.9 (86.5 to 90.0)	85.8 (83.7 to 86.8)
	Mortality	0.63 (0.49 to 1.04)	1.14 (0.9 to 1.8)
	**Harms (Nb of tests and treatment)**		
	Self-HPV test	11.02 (10.81 to 11.15)	7.11 (7.00 to 7.18)
	PAP test	1.44 (1.01 to 2.04)	0.97 (0.69 to 1.36)
	Surveillance (Self-HPV)	1.83 (1.28 to 2.70)	1.23 (0.87 to 1.80)
	PAP + HPV test after conization	0.15 (0.13 to 0.16)	0.13 (0.11 to 0.14)
	*Colposcopy*	0.76 (0.56 to 1.24)	0.57 (0.41 to 0.89)
	*Conization*	0.15 (0.13 to 0.16)	0.13 (0.11 to 0.14)
	*Total Visits*	2.51 (1.92 to 3.51)	1.80 (1.41 to 2.44)
**PAP/HPV**	**Benefits**		
	% of detected cancers	77.3 (76.4 to 77.4)	77.9 (75.9 to 78.9)
	% of detected cancers before 55 years	86.4 (82.9 to 88.8)	83.9 (81.0 to 86.0)
	Mortality	1.19 (0.77 to 2.35)	2.05 (1.39 to 3.61)
	**Harms (Nb of tests and treatment)**		
	PAP test	11.24 (10.38 to 11.37)	7.23 (6.81 to 7.29)
	HPV test	0.45 (0.17 to 1.63)	0.30 (0.12 to 1.07)
	Surveillance (PAP test alone)	0.91 (0.30 to 4.60)	0.60 (0.20 to 3.02)
	PAP + HPV test after conization	0.13 (0.10 to 0.14)	0.10 (0.08 to 0.12)
	*Colposcopy*	0.73 (0.36 to 3.36)	0.50 (0.26 to 2.23)
	*Conization*	0.13 (0.10 to 0.14)	0.10 (0.08 to 0.12)
	*Total Visits*	13.13 (12.26 to 18.6)	8.54 (7.95 to 12.28)

95% Credible Intervals are reported in brackets.

When the frequency of scheduled screening tests increased from 3 years to 5 years, the number of screening tests per woman was reduced by 40 %, total visits dropped from 30 % to 35 % and the number of colposcopies was also decreased by 25 % to 30 %. The number of conizations remained stable with the change of screening frequency. The percentage of detected cancers in a women’s lifetime was higher and the cervical cancer related mortality (per 1000 women) was 2-fold higher.

Overall, a more frequent rescreening frequency (3-years vs 5-years) and cytology screening strategies were associated with a greater number of lifetime total tests.

With the PAP/HPV strategy, the number of lifetime total visits were over 5-fold greater than with the Self-HPV strategies every 3 years. Indeed, with PAP/HPV, women had 13.13 visits in a lifetime, while with Self-HPV they had less than three visits. As for the number of conizations, women had almost the same number (0.13–0.16) with the three strategies at 3 years frequency in a lifetime. The number of colposcopies was higher with the Self-HPV/colpo strategy but it was similar for the Self-HPV/PAP and PAP/HPV strategies.

**Fig. 1:**
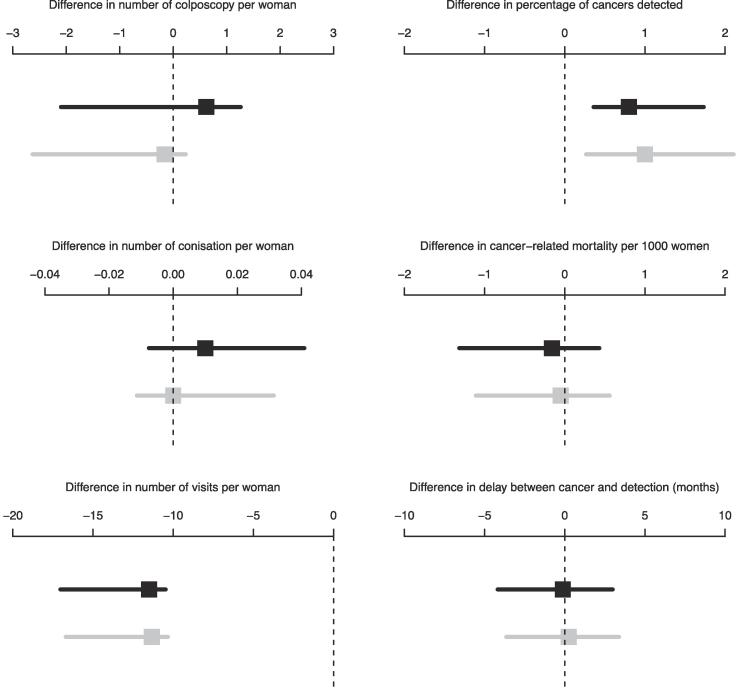
Distribution of different harm and benefit outcomes according to different strategies (Self-HPV/colpo and Self-HPV/PAP every 5 years compared with PAP/HPV every 3 years). Self-HPV/colpo every 5 years vs PAP/HPV every 3 years (black squares). Self-HPV/PAP every 5 years vs PAP/HPV every 3 years (grey squares). Squares represent mean differences and horizontal lines represent 95 % credible intervals.

Fig. 1 represents the distribution of different harm and benefit outcomes according to different strategies (Self-HPV/colpo and Self-HPV/PAP every 5 years versus PAP/HPV every 3 years). Squares represent mean differences and vertical lines represent 95 % credible intervals. The number of cancers detected was higher with Self-HPV/colpo every 5 years compared to 3-years PAP/HPV (mean difference: 0.80, 95 % CI: 0.36 to 1.73) and the mortality was lower for 5-years Self-HPV/colpo (mean difference: −0.16, 95 % CI: −1.32 to 0.43) and 5-years Self-HPV/PAP (mean difference: −0.05, 95 % CI: −1.11 to 0.56) vs 3-years PAP/HPV. On the other hand, the number of total visits was 11-fold higher with the PAP/HPV strategy compared to the Self-HPV screening strategies (mean differences (95 %CI): −11.5 (-17.0 to −10.5) for Self-HPV/colpo and −11.3 (-16.7 to −10.3) for Self-HPV/PAP). The number of conisations was similar with Self-HPV/PAP and PAP/HPV, but higher with Self-HPV/colpo.

## 4 Discussion

This modelling study compared the harms and benefits of cervical cancer screening between three screening strategies. We found that a woman's lifetime risk of contracting cancer or dying from cancer was lower with Self-HPV screening strategies than with cytology-based screening, not only when the tests were performed every 3 years, but also when Self-HPV was performed every 5 years compared to cytology every 3 years. In our opinion, the balance between harms and benefits of Self-HPV remained acceptable for a screening strategy while taking into account the greater reduction in mortality in the non-screened population.

All modelled screening strategies were estimated to result in reductions in cervical cancer cases and deaths and gains in life-years. The percentage of detected cervical cancers during a lifetime was around 77 % when the cancers appeared between the ages of 25 years and 55 years and 68 % for cancers occurring after 55 years. Mortality in women with cancer detected after 45 years was still lower when screened, which indicates that screening is useful, even if women start at a later age. Moreover, screening allowed an earlier detection of cervical cancer with all strategies, especially the Self-HPV strategies, which resulted in a higher detection of cervical cancer in the early stages of the disease (FIGO 1). Early diagnosis and treatment is critical to reduce the disease burden, since advanced cervical cancer is associated to higher mortality rates and treatment costs (Denny, 2012). Self-HPV strategies also allowed an early detection of cancers in terms of age compared to cytology, which resulted in a lower overall percentage of cervical cancer in a women’s lifetime.

One of the harms assessed by the model was the total number of visits in a lifetime. Self-HPV strategies considerably decreased the number of clinical visits compared to the classic cytological screening. As investigated before (Catarino et al., 2016), practical barriers are foremost to cervical cancer screening participation in Switzerland, such as lack of time and the cost of screening. Cervical cancer prevention requires multiple visits for screening, diagnosis, treatment, and follow-up, which is time consuming and associated with elevated costs for the patient. A reduction in the overall number of visits might therefore have an impact in cervical cancer screening compliance by eliminating those barriers.

Our results are in line with previous published studies performed worldwide (Farnsworth et al., 2020, Holt et al., 2020, Kim et al., 2018, Loopik et al., 2021, Sawaya et al., 2019, Thomsen et al., 2021, Zorzi et al., 2013). In American studies (Holt et al., 2020, Kim et al., 2018, Sawaya et al., 2019), cytology-based screening every 3 years had the lowest benefit in terms of life-years gained and prevented cervical cancer cases, though the total number of colposcopies was smaller. Cytology also had the highest level of harm, when taking into account the total number of tests. Overall, these studies reported that undergoing a cytology exam every 3 years from age 21 years or switching to a low-cost high-risk HPV test every 5 years from age 30 to 65 years presented a good balance of benefits and harms. Co-testing seemed to be an inefficient strategy compared to strategies involving HPV testing alone. European studies (Loopik et al., 2021, Thomsen et al., 2021, Zorzi et al., 2013) also showed that HPV primary screening provided an increased cervical cancer detection compared to cytology but at the price of increased colposcopy referrals. However, as shown in our analysis, by increasing the frequency of screening, the number of referrals was decreased while the screening benefits remained similar.

Debate regarding the best triage method for HPV positive women is ongoing (Cuschieri et al., 2018). Most countries use cytology as a triage test and our results suggest that it provides an excellent balance of harms and benefits, whereas primary HPV testing is performed every 5 years. The Self-HPV/PAP strategy performed every 5 years not only had a dramatically lower number of total visits than PAP/HPV (1.80 versus 13.13), it also had a lower number of colposcopies (0.57 versus 0.73), while having similar cancer related mortality. The number of conizations remained similar for each screening strategy.

Self-HPV/colpo has also been identified as an efficient cost-effective strategy (Vassilakos et al., 2019). The choice between Self-HPV and Self-HPV/colpo depends on a threshold ratio that would be considered a reasonable balance of harms and benefits. Mortality was the lowest at any frequency of screening for Self-HPV/colpo at the expense of a higher number of colposcopies. However, the desired cut-off was not clear when using colposcopies or total number of tests as a proxy for harm. Therefore, Self-HPV/colpo might be a suitable strategy for countries like Switzerland.

This study reports the first evaluation of the harms and benefits for cervical cancer screening with HPV testing in Switzerland. We used data input from the most recent prospective studies and modelled the present management of abnormal test results with high fidelity.

### 4.1 Limitations

This study also has some limitations. First, the analysis was based on assumptions of an independence of compliance at a screening step based on compliance with the previous one. A compliance rate at each screening procedure was assumed, however some women may refuse to participate in cervical cancer screening throughout their whole life. This type of behaviour was not modelled owing to lack of data. Another limitation previously mentioned (Vassilakos et al., 2019) is that we used HPV-testing starting at the age of 25 years. Some researchers suggest that HPV primary screening should start at the age of 30 years, but we focused on a specific population of women who did not participate in cervical cancer screening by offering a cost-effective test. Moreover, we did not take into account HPV vaccination, which may have an important impact in cervical cancer screening. Therefore, our findings are valid for unvaccinated women. HPV vaccination has been recommended in Switzerland since 2007 for women aged between 11 and 26 years (Office fédéral de la santé publique, 2015). Data from Swiss cross-sectional surveys have shown that HPV vaccine had no effect in never and under cervical cancer screening tendency (Jolidon et al., 2020). Another limitation is that some types of harm are difficult to assess, including the frequency of screening itself (3 to 5 years), which sometimes requires patients’ preference. Some women might feel more anxious regarding longer screening frequency and this might be taken into account, especially in countries with opportunistic screening like Switzerland (Catarino et al., 2016, Vassilakos et al., 2019), where shared decision making is necessary and useful.

## 5 Conclusions

Finally, in order to ensure systematic screening and follow-up of abnormal results, efforts should be directed towards the transition from an opportunistic to an organized screening programme in Switzerland, including primary HPV screening, which will ultimately lead to a better prevention of cervical cancer. Self-HPV should be offered as a complement according to each woman’s preference. Adjustments of the screening guidelines, including HPV triage testing are warranted to avoid excessive colposcopy referrals, which may cause avoidable distress to women.

In this study, we identified that Self-HPV screening strategies seem to show a reasonable balance of harms and benefits when performed every 5 years compared with cytology every 3 years. When using colposcopy as a proxy for harms, Self-HPV/PAP might be most cost-effective with a better ratio of harms and benefits.

## CRediT authorship contribution statement

**Rosa Catarino:** Conceptualization, Writing - original draft, Writing - review & editing. **Pierre Vassilakos:** Validation, Conceptualization, Writing - review & editing. **Patrick Petignat:** Writing – review & editing. **Christophe Combescure:** Methodology, Formal analysis, Supervision.

## Declaration of Competing Interest

The authors declare that they have no known competing financial interests or personal relationships that could have appeared to influence the work reported in this paper.

## Data Availability

Data will be made available on request.
